# Hyponastic Leaves 1 Interacts with RNA Pol II to Ensure Proper Transcription of MicroRNA Genes

**DOI:** 10.1093/pcp/pcad032

**Published:** 2023-04-11

**Authors:** Dawid Bielewicz, Jakub Dolata, Mateusz Bajczyk, Lukasz Szewc, Tomasz Gulanicz, Susheel S Bhat, Anna Karlik, Monika Jozwiak, Artur Jarmolowski, Zofia Szweykowska-Kulinska

**Affiliations:** Department of Gene Expression, Institute of Molecular Biology and Biotechnology, Faculty of Biology, Adam Mickiewicz University, Poznan 61-614, Poland; Centre for Advanced Technologies, Adam Mickiewicz University, Poznan 61-614, Poland; Department of Gene Expression, Institute of Molecular Biology and Biotechnology, Faculty of Biology, Adam Mickiewicz University, Poznan 61-614, Poland; Department of Gene Expression, Institute of Molecular Biology and Biotechnology, Faculty of Biology, Adam Mickiewicz University, Poznan 61-614, Poland; Department of Gene Expression, Institute of Molecular Biology and Biotechnology, Faculty of Biology, Adam Mickiewicz University, Poznan 61-614, Poland; Department of Gene Expression, Institute of Molecular Biology and Biotechnology, Faculty of Biology, Adam Mickiewicz University, Poznan 61-614, Poland; Centre for Modern Interdisciplinary Technologies, Nicolaus Copernicus University, Torun 87-100, Poland; Department of Gene Expression, Institute of Molecular Biology and Biotechnology, Faculty of Biology, Adam Mickiewicz University, Poznan 61-614, Poland; Department of Gene Expression, Institute of Molecular Biology and Biotechnology, Faculty of Biology, Adam Mickiewicz University, Poznan 61-614, Poland; Department of Gene Expression, Institute of Molecular Biology and Biotechnology, Faculty of Biology, Adam Mickiewicz University, Poznan 61-614, Poland; Department of Gene Expression, Institute of Molecular Biology and Biotechnology, Faculty of Biology, Adam Mickiewicz University, Poznan 61-614, Poland; Department of Gene Expression, Institute of Molecular Biology and Biotechnology, Faculty of Biology, Adam Mickiewicz University, Poznan 61-614, Poland

**Keywords:** *Arabidopsis thaliana*, DRB1, HYL1, MicroRNA, Transcription

## Abstract

Hyponastic Leaves 1 (HYL1) [also known as Double-stranded RNA-Binding protein 1 (DRB1)] is a double-stranded RNA-binding protein involved in microRNA (miRNA) processing in plants. It is a core component of the Microprocessor complex and enhances the efficiency and precision of miRNA processing by the Dicer-Like 1 protein. In this work, we report a novel function of the HYL1 protein in the transcription of miRNA (*MIR)* genes. HYL1 colocalizes with RNA polymerase II and affects its distribution along *MIR* genes. Moreover, proteomic experiments revealed that the HYL1 protein interacts with many transcription factors. Finally, we show that the action of HYL1 is not limited to *MIR* genes and impacts the expression of many other genes, a majority of which are involved in plastid organization. These discoveries indicate HYL1 as an additional player in gene regulation at the transcriptional level, independent of its role in miRNA biogenesis.

## Introduction

MicroRNAs (miRNAs) are small ∼21-nt-long RNA molecules that play a vital role in the post-transcriptional regulation of gene expression (Bartel 2004). miRNAs are involved in a vast range of metabolic processes and hence act as regulatory molecules in overall plant development and in the plant response to environmental conditions (Palatnik et al. 2003, Chen 2004, Kruszka et al. 2012, Barciszewska-Pacak et al. 2015). miRNA genes (*MIR*) are transcribed by RNA polymerase II (Pol II) (Xie et al. 2005), and as in the case of protein-coding genes, they are regulated at the transcriptional and post-transcriptional levels in a similar way. For example, changes in the phosphorylation of the RNA Pol II C-terminal domain (CTD) by Cyclin-Dependent Kinase Ds and Cyclin-Dependent Kinase F;1 have been reported to modulate transcription, co-transcriptional capping, polyadenylation and splicing of all RNA Pol II transcripts (Hajheidari et al. 2012, 2013). The transcription coactivator complex Mediator plays a general role in recruiting Pol II to gene promoters during transcription initiation (Kim et al. 2011). *MIR* gene transcription and protein-coding gene transcription are modulated by the Carbon Catabolite Repression 4–Negative on TATA-less (NOT) complex subunit NOT2, the Elongator complex subunits ELP2 and ELP5, the MYB-R2R3-type transcription factor cell division cycle 5 (CDC5) and the DNA-binding with one finger (DOF) transcription factor cycling DOF factor 2 (Wang et al. 2013, Zhang et al. 2013, Fang et al. 2015, Sun et al. 2015). However, a common feature of all primary transcripts of *MIR* genes [known as primary miRNAs (pri-miRNAs)], which is not necessary for protein-coding gene transcription, is the formation of a stem-loop region. In this stem-loop region, mature miRNA is embedded. Mature miRNAs are released from pri-miRNAs by a series of cleavages performed by a protein complex called the Microprocessor. Notably, the stem-loop region of pri-miRNAs serves as a mark for the Microprocessor to recognize and start processing the pri-miRNAs (Kurihara and Watanabe 2004, Mateos et al. 2010, Zhu et al. 2013, Dolata et al. 2018). The core of the Microprocessor complex is built of three proteins, Dicer-Like 1 (DCL1), Double-stranded RNA-Binding protein 1 (DRB1) [also known as Hyponastic Leaves 1 (HYL1)] and Serrate (SE) (Yang et al. 2006, Fang and Spector 2007, Dong et al. 2008, Dolata et al. 2018). DCL1 is a RNAse III-type enzyme that cleaves pri-miRNAs in multiple places to eventually facilitate the release of mature miRNA/mirRNA* duplexes (Park et al. 2002, Reinhart et al. 2002, Kurihara et al. 2006, Bologna et al. 2013). HYL1 and SE assist in increasing the accuracy of pri-miRNA cleavage by DCL1 (Kurihara et al. 2006). All three of these core components are necessary for proper functioning of the Microprocessor, and in hypomorphic mutants of DCL1 and SE, and null mutants of HYL1, pri-miRNAs accumulate, and mature miRNAs are downregulated (Laubinger et al. 2008, Szarzynska et al. 2009, Zielezinski et al. 2015). HYL1 contains two double-stranded RNA-binding domains (dsRBDs) in its N-terminal region followed by a nuclear localization signal (NLS) and an unstructured C-terminal region (Yang et al. 2010). HYL1 is considered to form a homodimer and bind the miRNA/miRNA* duplex region of pri-miRNA (Burdisso et al. 2014, Yang et al. 2014). Knock-out of HYL1 has a pleiotropic impact on the phenotype of plants (among the other defects in leaf shape and late flowering time) and their response to auxin and ABA (Lu and Fedoroff 2000, Vazquez et al. 2004). To date, no ortholog of HYL1 has been identified in mammalian cells. However, in the cnidarian *Nematostella vectensis*, a ‘plant-like’ HYL1 homolog was identified (Tripathi et al. 2022). Cnidarian HYL1 protein is involved in miRNA biogenesis; however, it interacts only with pre-miRNAs and not with pri-miRNAs. Additionally, mammalian RNA nucleases involved in miRNA biogenesis, namely, Drosha and Dicer, are also required for proper functioning with the assistance of proteins containing dsRBDs, namely, DiGeorge syndrome critical region 8 (DGCR8) and transactivation response element RNA-binding protein (TRBP), respectively (Han et al. 2004, Chendrimada et al. 2005, Haase et al. 2005). Both HYL1 and DGCR8 are nuclear proteins involved in the first steps of pri-miRNA maturation, i.e. cleavage by RNAse III (Gregory et al. 2004). However, similar to TRBP, HYL1 can be phosphorylated, which affects its activity (Paroo et al. 2009, Manavella et al. 2012, Achkar et al. 2018).

MiRNA production in mammalian cells is co-transcriptional, as Drosha and DGCR8 have been shown to bind regions in the proximity of many gene promoters (not only *MIR* genes), and consequently, Drosha knockdown in HeLa cells negatively impacts general gene transcription (Gromak et al. 2013). Similarly, in plants, co-transcriptional processing of pri-miRNAs has been proposed to occur through the association of DCL1 with the chromatin, Mediator and Elongator complex (Fang et al. 2015, Cambiagno et al. 2021). Co-transcriptional processing is supported by the fact that transcription of *MIR* loci is required for the association of DCL1 with the chromatin and Elongator complex (Fang et al. 2015). Additionally, the DCL1 interaction with *MIR* loci is modulated by Hasty (HST) protein in a Mediator-dependent manner (Cambiagno et al. 2021). HST interacts with MED37 (Mediator complex protein) and acts as a scaffold to further stabilize the MED37-DCL1 complex and allow the assembly of the Microprocessor complex early on in pri-miRNA transcription. Moreover, co-transcriptional processing of pri-miRNAs also depends on whether a given pri-miRNA is processed from loop-to-base or base-to-loop, a process that also involves PRP40A protein (Gonzalo et al. 2022, Stepien et al. 2022). Consistent with these ideas and since the interaction between DCL1, HYL1 and *MIR* loci has been established (Kurihara et al. 2006, Cambiagno et al. 2021, Gonzalo et al. 2022), we asked whether HYL1 could be involved in the transcriptional regulation of *MIR* genes and may interact with the RNA Pol II complex.

## Results

### HYL1 is important for proper *MIR* gene transcription

**Fig. 1:**
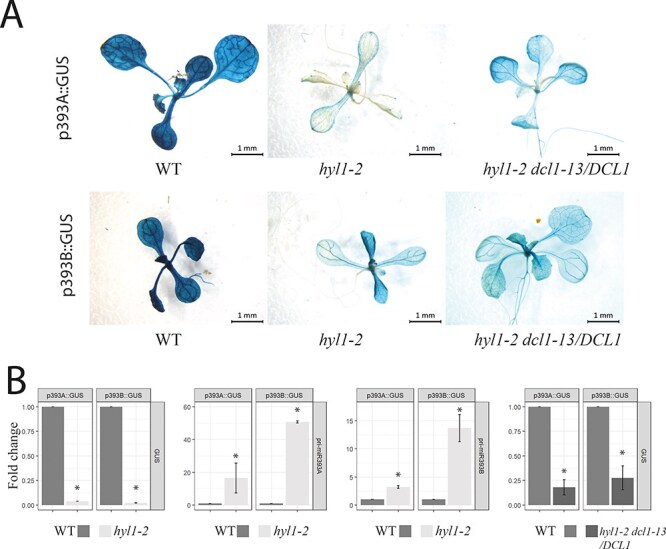
HYL1 is a positive factor stimulating the transcription of *MIR* genes. (A) GUS staining of seedlings representing reporter lines in WT (left), *hyl1-2* (middle) and *hyl1-2 dcl1-13/DCL1* (right) backgrounds. (B) RT-qPCR analysis of GUS transcript and pri-miRNA levels in the reporter lines in WT, *hyl1-2* and *hyl1-2 dcl1-13/DCL1* backgrounds. Error bars indicate SD (*n* = 3). Asterisk represents the statistically significant fold change of the indicated sample compared to the WT sample (*P* < 0.05).

To investigate the possibility that the HYL1 protein is involved in the transcription of *MIR* genes, we used a GUS reporter line system. We independently crossed two reporter lines with GUS under two different *MIR* gene promoters [p*MIR393A*:GUS and p*MIR393B*:GUS (Parry et al. 2009)] in the wild-type (WT) [Columbia-0 (Col-0)] background with *hyl1-2* mutants (HYL1 knock-out mutants). We observed that the expression of the GUS protein driven by *MIR* gene promoters was markedly lower in the *hyl1-2* background than in Col-0 (**Fig. 1A**). Using RT-qPCR, we also measured the level of GUS transcripts in p*MIR393A*:GUS, p*MIR393B*:GUS, p*MIR*393A:GUSx*hyl1-2* and p*MIR*393B:GUSx*hyl1-2* reporter lines and observed a significant decrease in the level of GUS transcripts in p*MIR*393A:GUSx*hyl1-2* and p*MIR*393B:GUSx*hyl1-2* plants (**Fig. 1B**). Moreover, in the absence of HYL1 protein, pri-miRNAs are not processed into pre-miRNA and finally into mature miRNAs. Using RT-qPCR, we observed a significant increase in the level of endogenous pri-miRNA393A and pri-miRNA393B in p*MIR*393A:GUSx*hyl1-2* and p*MIR*393B:GUSx*hyl1-2* reporter lines, respectively (**Fig. 1B**, middle part). The downregulation of GUS transcripts suggested that HYL1, which is involved in pri-miRNA processing, may also regulate the transcription of *MIR* genes. However, it is also possible that the strong downregulation of GUS expression in the *hyl1-2* background is caused by a feedback mechanism in which the global downregulation of miRNAs in *hyl1-2* results in upregulation of as-yet-unidentified transcriptional factors that in turn inhibit *MIR393A* and *MIR393B* transcription. To address this question, we decided to restore the miRNA levels in the reporter lines within the *hyl1-2* background. For this purpose, we crossed p*MIR*393A:GUSx*hyl1-2* and p*MIR*393B:GUSx*hyl1-2* mutant plants with a *DCL1* mutant: namely, *hyl1-2 dcl1-13/dcl1-13* (homozygous *hyl1-2* suppressor) (Tagami et al. 2009). The *hyl1-2 dcl1-13/dcl1-13* mutant has a point mutation in the *DCL1* gene that promotes DCL1 activity in the absence of HYL1. The point mutation in the *DCL1* gene results in an amino acid substitution of Glu to Lys in the ATPase/DExH-box RNA helicase domain. Hence, the expression of the *dcl1-13* allele in the *hyl1-2* background restores the level of miRNAs and rescues plants from the developmental abnormalities associated with low levels of miRNAs (Tagami et al. 2009). We prepared heterozygous *hyl1-2* suppressor (namely, *hyl1-2 dcl1-13/DCL1*) transgenic plants carrying the genomic sequence of the mutated *DCL1* gene under its native promoter. We obtained four independent transgenic lines and confirmed previous results, showing that the expression of the *dcl1-13* allele restores mostly the *hyl1-2* phenotype. However, the suppressor effect was mild, and the levels of pri-miRNAs and mature miRNAs in the *hyl1-2* background were only partially restored (**Supplementary Fig. S1**). We then crossed our reporter lines (p*MIR*393A:GUS and p*MIR*393B:GUS) with *hyl1-2 dcl1-13/DCL1* transgenic plants. Analysis of GUS staining in the offspring clearly showed that the GUS protein is still inefficiently expressed in the *hyl1-2 dcl1-13/DCL1* mutant background (**Fig. 1A**, right panel). Additionally, we also performed RT-qPCR analysis to measure the GUS transcript level, and as in the *hyl1-2* mutant, in *hyl1-2 dcl1-13/DCL1* mutant plants, the GUS transcript was downregulated in comparison to the expression in WT plants (**Fig. 1B**, right panel). Moreover, to exclude the possible feedback mechanism or general post-transcriptional gene silencing perturbation, we also crossed our reporter line with the *hen1-5* mutant. GUS staining in the offspring showed that GUS protein is expressed at a similar level in *hen1-5* and in WT backgrounds (**Supplementary Fig. S2**). Hence, our data support the idea that HYL1 is a direct positive regulator of *MIR* gene transcription. To exclude the possibility that the observations were specific to the miR393 family, we crossed four additional GUS reporter lines with the *hyl1-2* mutant. We observed, like previously, that the expression of the GUS protein driven by *MIR* gene promoters was lower in the *hyl1-2* background in comparison to the WT background (**Supplementary Fig. S3**).

### The HYL1 protein interacts with transcription factors

**Fig. 2:**
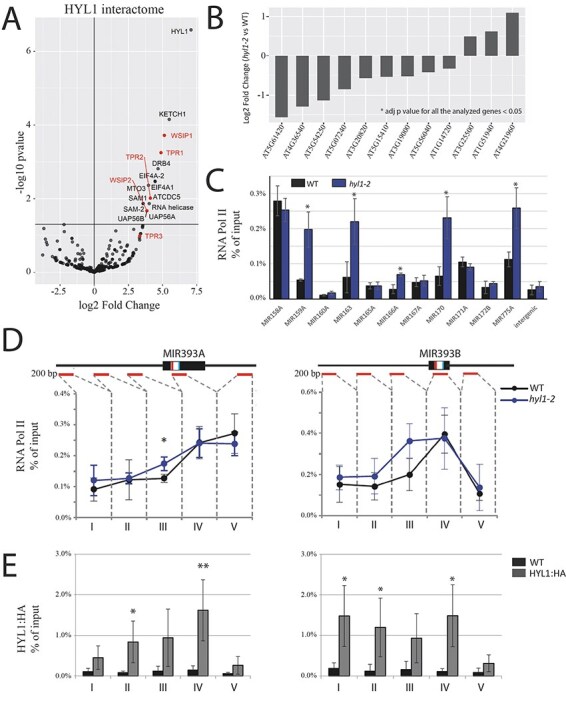
HYL1 interacts with transcription factors and is important for the proper distribution of RNA Poly II along *MIR* genes. (A) HYL1 interactome determined by co-IP followed by mass spectrometry. (B) The comparison of the expression levels of 12 TPR1 target genes in WT and *hyl1-2* plants. The adjusted *P*-value (DESeq2 test; Love et al. 2014) for all the tested genes was <0.05. (C) The occupancy of total RNA Pol II in promoter regions on different *MIR* genes (∼200 bp upstream of TSS) was determined by ChIP followed by qPCR. (D) The occupancy of total RNA Pol II at the *MIR393A* or *MIR393B* genes was determined by ChIP followed by qPCR. The region marked with an asterisk represents the statistically significant enrichment of RNA Pol II in *hyl1-2* compared to WT plants. A schematic of the gene structure is shown, and the lines below the gene structures show the amplified regions. Error bars represent the SD of three independent biological replicates. (E) The accumulation of HYL1 in the HYL1:HA mutant and WT plants at the *MIR393A* or *MIR393B* genes determined by ChIP followed by qPCR.

Since we found that HYL1 acts as a positive regulator of GUS transcription under the *MIR* gene promoter, we decided to test whether it interacts with transcription factors. For this experiment, we used a transgenic line containing HYL1 tagged with the hemagglutinin (HA) epitope (*pHYL1:HYL1:HA*) and anti-HA antibodies to coimmunoprecipitate HYL1 with its potential protein interactors, which were subsequently identified by mass spectrometry analysis. As a benchmark for our experiment, we searched for previously published data regarding HYL1 interactors and compared them with our data. We were able to identify bona fide HYL1 partners karyopherin enabling the transport of the cytoplasmic HYL1 (KETCH1) and CDC5 in our data (**Fig. 2A**) (Zhang et al. 2013, 2017). KETCH1 is a karyopherin enabling the import of HYL1 to the nucleus, and CDC5 is an MYB transcription factor. Interestingly, CDC5 was shown to be involved in the transcription of *MIR* genes (Zhang et al. 2013). Similar to *hyl1-2* mutant plants, in the CDC5 knock-out mutant, the GUS reporter, which was under the control of the *MIR* gene promoter, was downregulated (Zhang et al. 2013). However, *cdc5* mutants showed no enrichment at the pri-miRNA level, which can be explained by the fact that CDC5 is involved only in the transcription of *MIR* genes, not in the processing of pri-miRNA, similar to HYL1 (Vazquez et al. 2004, Zhang et al. 2013). Interestingly, in our coimmunoprecipitation (co-IP) results, we found that HYL1 was also associated with transcription factors from the TOPLESS transcription factor family (**Fig. 2A**). This protein family contains five members, which act redundantly and mediate transcriptional repression (Zhu et al. 2010). All of the members from the TOPLESS family were found in the HYL1 co-IP assay. Zhu et al. showed that TPR1 was recruited to the promoters of 12 genes and repressed their expression. We analyzed the expression levels of these 12 genes in the *hyl1-2* background in comparison to WT plants using publicly available transcriptomic data (Manavella et al. 2012). We found that the expression of all identified TPR1 targets was significantly changed (adjusted *P* < 0.05, DESeq2 test) between WT and *hyl1-2* plants (**Fig. 2B**). Interestingly, the expression of nine of these targets was decreased in the *hyl1-2* background. This suggests that TPR1 is more active when HYL1 protein is absent and that the interaction of HYL1 with TPR1 is necessary to decrease TPR1 repression activity. However, the interaction between TOPLESS transcription factors and HYL1 requires a more detailed investigation.

### RNA Pol II occupancy on *MIR* and selected protein-coding genes is affected in the *hyl1-2* mutant

In light of our data showing that HYL1 might be involved in *MIR* gene transcription, we tested whether it affects RNA Pol II occupancy on *MIR* genes. For this purpose, we performed a chromatin immunoprecipitation (ChIP) assay using an anti-RNA Pol II CTD repeat YSPTSPS antibody (8WG16 clone) (henceforward referred to as antibodies against total RNA Pol II) followed by qPCR in WT and *hyl1-2* mutants. We compared the occupancy of RNA Pol II on selected *MIR* genes [∼200 base pairs (bp) upstream of the transcription initiation start site] in *hyl1-2* and WT plants (**Fig. 2C**). We selected the *MIR* genes that were tested previously in *cdc5* mutant plants (Zhang et al. 2013). The results showed that the RNA Pol II distribution is affected in several *MIR* gene promoter regions. Our data showed increased accumulation of RNA Pol II in the region ∼200-bp upstream from the transcription start site (TSS) for five tested *MIR* genes in the *hyl1-2* mutant compared to WT plants. This observation is opposite to what was reported in *cdc5* mutant plants (Zhang et al. 2013). Additionally, we examined the RNA Pol II distribution in detail in the *MIR393A* and *MIR393B* genes (**Fig. 2D**). Analysis showed that RNA Pol II accumulates in the region ∼200-bp upstream from the transcription initiation site. Keeping in mind our previous results where transcription of the GUS reporter was downregulated in a *hyl1-2* mutant compared to WT plants, this may suggest that the accumulation of RNA Pol II in the promoter region of *MIR* genes in *hyl1-2* is a result of RNA Pol II stalling. Furthermore, we performed ChIP-seq experiments using antibodies against total RNA Pol II in WT and *hyl1-2* mutant plants for a global analysis of RNA Pol II distribution along *MIR* genes. For the global ChIP-seq analysis, we selected only miRNAs that are encoded by independent transcriptional units with a previously determined TSS (Bielewicz et al. 2012, Zielezinski et al. 2015). The results show increased RNA Pol II occupancy in the region of the TSS as well as in the gene body of *MIR* genes in the *hyl1-2* mutant in comparison to WT plants (**Supplementary Fig. S4A**). Recently, similar to *hyl1-2* plants, the retention (increased level) of RNA Pol II in the gene body of *MIR* genes was also observed in PRP40, and it was demonstrated that PRP40 is involved in co-transcriptional processing of pri-miRNAs (Stepien et al. 2022). The ChIP-seq data also confirmed the results obtained for the *MIR*393A gene (**Supplementary Fig. S4B**). We also tested the possibility of HYL1 having a broader role in RNA Pol II transcription initiation. Using our ChIP-seq data, we analyzed 200 bp region upstream of the transcription initiation start of protein-coding genes. Analysis showed that around 60% of tested genes have higher RNA Pol II occupancy (**Supplementary Fig. S4C**). We next examined whether HYL1 binds to the promoter of *MIR* genes. We performed ChIP followed by qPCR, using an antibody against HA in the *hyl1-2* complementation line containing pHYL1:HYL1:HA and WT plants as a control. We examined the HYL1 distribution in detail in the *MIR393A* and *MIR393B* genes. We observed some enrichment of the tested fragments in the HYL1:HA complex (**Fig. 2E**). Moreover, we observed enrichment for the six tested *MIR* gene promoter in the region ∼200 bp upstream from the transcription initiation site in the HYL1:HA line (**Supplementary Fig. S5A**). In addition, we were curious if HYL1 can bind promoters of protein-coding genes. We randomly selected few genes (from the pool of 654 specifically downregulated genes in *hyl1-2* genes, as mentioned later) and tested if HYL1 can bind to the promoter regions of these genes. The results suggest that HYL1 can bind to promoter regions of the selected genes (**Supplementary Fig. S5B**). These results suggested that HYL1 is associated with *MIR* gene promoters, and this might suggest that HYL1 is a more general factor acting in transcription initiation of RNA Pol II.

### HYL1 affects the expression of many RNA Pol II genes

**Fig. 3:**
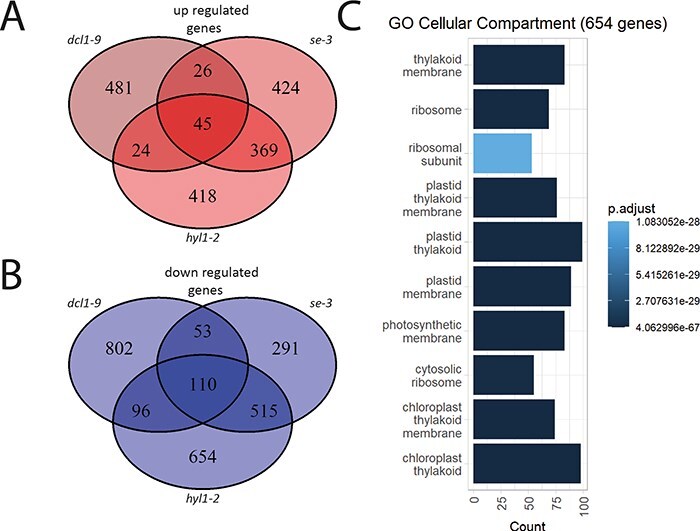
Downregulation of RNA Pol II transcripts in the HYL1 mutant. (A) A Venn diagram showing the overlap of significantly upregulated genes between *hyl1-2*, and *se-3* and *dcl1-9* mutants when compared to WT plants. (B) A Venn diagram showing the overlap of significantly downregulated genes between *hyl1-2*, and *se-3* and *dcl1-9* mutants when compared to WT plants. (C) Gene ontology analysis performed on 654 genes that are downregulated in the *hyl1-2* mutant and no change in the expression of *se-3* and *dcl1-*9 mutant plants.

We were interested in whether HYL1 could also affect the transcription efficiency of other genes transcribed by RNA Pol II. To investigate this possibility, we analyzed publicly available transcriptomic data obtained from WT and *hyl1-2* mutant plants (Manavella et al. 2012). Theoretically, in the *hyl1-2* mutant, a generally low level of miRNAs should be accompanied by a general upregulation of miRNA targets. We found that the expression of 2,231 genes was affected in the *hyl1-2* mutant compared to WT plants. A total of 1,375 (∼60%) of the differentially expressed genes (DEGs) were downregulated in the *hyl1-2* mutant compared to WT plants. We then compared DEG expression between *hyl1-2* and WT with the transcriptomic data obtained for the most severe hypomorphic mutant of the *SE* gene, *se-3* and *dcl1-9* mutants. Our analysis showed that out of the 856 upregulated genes in *hyl1-2*, 438 (>50%) were also upregulated in *se-3* or *dcl1-*9 (**Fig. 3A**). Among the 1,375 downregulated genes in *hyl1-2*, 721 (>50%) were upregulated in *se-3* or *dcl1-*9 (**Fig. 3B**). We are aware that the gene regulatory cascade altered by the miRNA reduction can potentially lead to different outcomes in various miRNA biogenesis mutants. However, we were curious if we can find differentially regulated genes whose expression is HYL1-dependent and SE- and DCL1-independent. We found a large group of DEGs that are specifically downregulated in *hyl1-2* mutant plants (654 genes). Analysis of our ChIP-seq data carried out in the *hyl1-2* mutant revealed that RNA Pol II occupancy on the TSS and gene body was markedly increased in the case of genes whose expression was downregulated only by HYL1 (pool of 654 genes) (**Supplementary Fig. S4D**). Gene ontology analysis showed that the proteins encoded by these genes function mostly in chloroplasts (**Fig. 3C**). Moreover, the largest group of genes showing downregulated expression in the *hyl1-2* mutant (∼60 genes) is involved in biological processes of ‘plastid organization’ (**Supplementary Fig. S6**), which suggest that HYL1 might be involved in light sensing and/or chloroplast development.

### HYL1 is associated with RNA Pol II

**Fig. 4:**
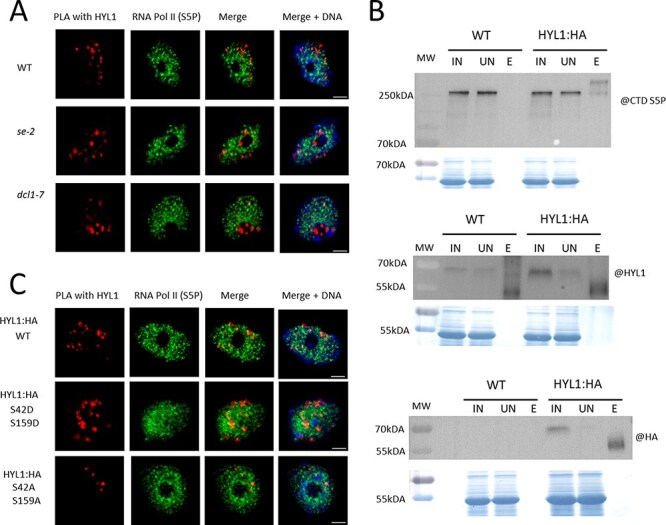
The interaction between HYL1 and RNA Pol II investigated by PLA and co-IP experiments. (A) Positive PLA signals (spots in the first column) can be seen in WT and se-2 and dcl1-7 backgrounds. RNA Pol II is represented in green. DNA is stained with Hoechst (blue). (B) Phosphorylated Ser2 of CTD can be detected by immunoprecipitation of HYL1 followed by Western blot. MW, a molecular weight marker; IN, an input fraction of proteins before the immunoprecipitation; UN, an unbound fraction of the proteins after the incubation with the indicated antibodies; E, a fraction of proteins eluted from the beads. The bottom panels represent amido black staining as a loading control. (C) Positive PLA signals can be seen mostly in cells expressing the native HYL1 (WT) and phosphomimickry HYL1 (S42D S159D) versions.

The observation that HYL1 is required for proper transcription of the GUS gene under the *MIR393A* and *MIR393B* promoters and that lack of HYL1 affects RNA Pol II occupancy at *MIR* gene promoters prompted us to test the stage at which transcription is affected. We performed initially immunolocalization in fixed nuclei from WT plants and observed strong colocalization of HYL1 and total RNA Pol II (**Supplementary Fig. S7**). We used antibodies specific for serine 5 (Ser5) (transcription initiation) and serine 2 (Ser2) (transcription elongation) of the RNA Pol II CTD independently to further identify the transcription steps at which colocalization takes place. The results suggest that HYL1 is already associated with RNA Pol II at the transcription initiation stage (Ser5) and remains associated with RNA Pol II during the elongation step (Ser2). Since HYL1 is a part of the Microprocessor core complex, we considered the possibility that HYL1 is brought to RNA Pol II by another member of the Microprocessor. Recently, it was shown that SE and DCL1 are directly associated with specific regions of *Arabidopsis* chromatin (Fang et al. 2015, Speth et al. 2018). In addition, SE is involved in many processes associated with RNA metabolism, such as splicing, 3ʹ-end formation, RNA transport and RNA stabilization (Laubinger et al. 2008, Raczynska et al. 2014). To exclude the possibility of SE-mediated colocalization of HYL1 and RNA Pol II, we investigated the colocalization of HYL1 with RNA Pol II in the *se-2* mutant plants (**Supplementary Fig. S8**). Similar to our previous experiment, the results were obtained for total RNA Pol II and for RNA Pol II phosphorylated at Ser5 or Ser2. We did not observe any decrease in colocalization of HYL1 and RNA Pol II in the *se-2* mutant, showing that the association of HYL1 with RNA Pol II at least does not depend on full-length SE protein. Moreover, we performed proximity ligation assay (PLA) to see if HYL1 can interact with RNA Pol II in WT, *se-2* and homozygous *dcl1-7* mutants. In all tested plant backgrounds, we observed positive signals, which indicates that the HYL1 interaction with RNA Pol II does not depend on DCL1 or SE protein (**Fig. 4A**, **Supplementary Fig. S9**). To validate the PLA results, we used WT, as a negative control, and pHYL1:HYL1:HA plants for co-IP experiments followed by Western blot analysis. The results show that the largest subunit of RNA Pol II (phosphorylated at Ser2 of the CTD domain) can be immunoprecipitated with HYL1 protein (**Fig. 4B**). To further characterize the interaction, we used the Fluorescence Resonance Energy Transfer coupled with Fluorescence Lifetime Imaging Microscopy approach. We transiently expressed HYL1 fused with the Red fluorescent protein and CTD domain of RNA Pol II (with NLS) fused with GFP in *Arabidopsis* cells (**Supplementary Fig. S10**). The interaction between PRP40A with CTD was used as a positive control, and Zinc finger CCHC domain-containing protein 8 (ZCCH8) acted as a negative control (Bajczyk et al. 2020, Stepien et al. 2022). However, in this experiment, we did not observe the interaction between HYL1 and CTD. This might suggest that the HYL1 interaction with RNA Pol II does not depend solely on the CTD domain and/or the interaction requires post-translational modifications of the CTD-like phosphorylation of Ser2.

From previous studies, it was known that HYL1 undergoes post-translational modifications, which affects its function and cellular localization (Manavella et al. 2012, Achkar et al. 2018). Dephosphorylated HYL1 is an active form of protein in pri-miRNA processing, and phosphorylated HYL1 (especially at serine 42) cannot interact with RNA and is mainly localized in the nucleus. Based on these results, we were wondering if the phosphorylation status of HYL1 could be a factor that distinguishes its roles in transcription and in the processing of pri-miRNAs. To test our hypothesis, we used transgenic lines expressing HYL1 where two serines (S42 and S159), the most important for the HYL1 function, were mutated to alanines (pHYL1:HYL1:AA:HA) or aspartic acids (pHYL1:HYL1:DD:HA). Mutations to alanines should mimic dephosphorylated HYL1 and to phosphorylated aspartic acids. We perform PLA experiments to see if HYL1 can interact with RNA Pol II and to test if this interaction depends on the phosphorylation status of HYL1. In plants expressing WT and phosphomimicry of HYL1, we observed many positive signals, which indicates that HYL1 interacts with RNA Pol II and diminished signal in cells expressing dephosphorylated HYL1 (**Fig. 4C**, **Supplementary Fig. S9**).

## Discussion

The HYL1 protein plays a major role in the processing of pri-miRNAs, facilitating the recruitment of DCL1 to the pre-miR stem-loop structure. It was also reported that in the *A. thaliana hyl1-2* mutant, in the case of intron-containing *MIR* genes, both forms of pri-miRNA (before and after splicing) accumulate at high levels, suggesting that the lack of the HYL1 protein affects the splicing of primary miRNA transcripts (Szarzynska et al. 2009). Therefore, it seems that HYL1 may play additional roles in miRNA biogenesis. It appears that the recruitment of HYL1 to the miRNA biogenesis machinery takes place at the very early stages of pri-miRNA processing, possibly before splicing occurs. For protein-coding genes, a direct connection between gene transcription and further co- and post-transcriptional processing, including splicing of nascent pre-mRNAs, has been shown (Bauren and Wieslander 1994, Li et al. 2020). To our knowledge, all plant *MIR* genes analyzed to date are transcribed by RNA Pol II (Xie et al. 2005). In addition, similar to pre-mRNAs, primary transcripts of *MIR* genes undergo further processing, including cap structure formation, polyadenylation, splicing and m^6^A methylation (Xie et al. 2005, Bielewicz et al. 2013, Schwab et al. 2013, Knop et al. 2017, Bhat et al. 2020).

The results presented in this paper show that a GUS reporter under the control of an *MIR* gene promoter is expressed at a lower level in the absence of HYL1 (*hyl1-2* and *hyl1-2/dcl1-13* background). Moreover, the evaluation of the occupancy of RNA Pol II on *MIR* genes in *hyl1-2* showed an accumulation of total RNA Pol II in the region ∼200-bp upstream of the transcription initiation site. This phenomenon was observed in the case of *MIR393A* and *MIR393B*, as well as *MIR159A, MIR163, MIR166A, MIR170* and *MIR775A*. While the distribution of RNA Pol II in a variety of complex genomes is correlated with gene expression, the presence of RNA Pol II at a specific locus does not necessarily indicate active expression of this locus. The higher occupancy of RNA Pol II in the analyzed *MIR* promoter regions, combined with results from the GUS reporter assays, suggests that HYL1 is a positive factor of RNA Pol II transcription. This suggestion is supported by the results showing that HYL1 is associated with total RNA Pol II and that the association does not depend on DCL1 and SE proteins. It is known that the phosphorylation status of the CTD of RNA Pol II is very important in transcription (Komarnitsky et al. 2000), and the interplay between kinases and phosphatases acting on RNA Pol II modifies gene expression. One of the proteins that are able to dephosphorylate the CTD of RNA Pol II at the Ser5 residue is a protein phosphatase called CPL1 (Koiwa et al. 2004). Similarly, it was reported that the phosphorylation status of HYL1 is also important in miRNA biogenesis (Mendoza et al. 2005, Manavella et al. 2012, Raghuram et al. 2015, Su et al. 2017, Yan et al. 2017, Achkar et al. 2018, Bhagat et al. 2022). It was shown that the HYL1 protein needs to be dephosphorylated for optimal activity, and this activity is also maintained by the CPL1 protein. Fully phosphorylated HYL1 that is exclusively localized in the nucleus is not active in pri-miRNA processing; however, it continues to participate in the transcription of *MIR* genes. More importantly, the phosphorylation status of HYL1 could be a factor that distinguishes its roles in transcription and in the processing of pri-miRNAs. An alternative model suggests that the C-terminal region of HYL1, which displays a tendency to bind dsDNA, may interact with chromatin (Bhagat et al. 2018). We also observed that HYL1 interacts with transcriptional factors whose role is not limited to the regulation of *MIR* genes. For example, TPR1, the transcriptional corepressor that belongs to the TOPLESS gene family, binds to the promoter region of the selected protein-coding genes to decrease their expression (Zhu et al. 2010). We observed that the expression of these selected protein-coding genes was mostly downregulated (9 of 12 examples) in the *hyl1-2* mutant plants. This observation suggests that the interaction between HYL1 and TPR1 may inhibit the activity of the TPR1 protein. Taking into account that the expression of 1531 genes is downregulated in *hyl1-2* mutant plants, it is possible that the interaction of HYL1 with another TOPLESS family member can influence the expression of different sets of genes. Moreover, TPR1 is also an interesting example that connects the transcriptional regulation of protein-coding genes and the *MIR* genes. It was shown that in plants that overexpress the TPR1 protein, the level of pri-miRNAs is downregulated in comparison to that in WT plants (Cai et al. 2018). Our data present new possibilities regarding HYL1’s role in plant cells. Recently, in co-IP experiments performed in HYL1-Yellow fluorescent protein plants, Hou et al. found that HYL1 interacts with histone proteins and also with the U2 snRNP component, which supports our hypothesis that HYL1 is involved in the early steps of transcription (Hou et al. 2021). Moreover, the authors discovered that HYL1 interacts with many chloroplast important proteins, supporting the idea that HYL1 is involved in light sensing and/or chloroplast development. It is known that light is an important factor for HYL1 maintenance in the cell (Cho et al. 2014). At night, HYL1 is degraded by HYL1-CLEAVAGE SUBTILASE 1 protease, which results in the downregulation of mature miRNA levels (Jung et al. 2022). However, our analysis suggests that HYL1 may be involved in the plant response to light independent of its role in the miRNA pathway. Our data regarding the roles of HYL1 other than those in miRNA biogenesis are further supported by a recently published work that uncovered a novel function of HYL1 related to skotomorphogenesis (Sacnun et al. 2020). However, more work is needed to propose a detailed mechanism of how HYL1 affects transcription.

## Materials and Methods

### Plant material and growth conditions

*Arabidopsis thaliana* ecotype Col-0 plants were grown as WT plants, the insertion mutant SALK_064863 was grown as a knock-out mutant of HYL1 (*hyl1-2*), the insertion mutant SALK_049197 was grown as a knock-out mutant of HEN1 (*hen1-5*) and the insertion mutant SAIL_44_G12 was grown as a knock-out mutant of *se-2.* RNA-seq data also include expression data from the *se-3* mutant, which was the T-DNA insertion mutant SALK_083196 (Grigg et al. 2005). The *pMIR393A:GUS* and *pMIR393B:GUS* reporter lines that were used in this study were described previously (Parry et al. 2009). The *hyl1-2 dcl1-13/DCL1*, p*HYL1:HYL1:HA*, p*HYL1:HYL1:AA:HA* and p*HYL1:HYL1:DD:HA* transgenic lines were obtained by floral dip transformation of *hyl1-2* mutant plants (CLOUGH and BENT 1998). Genomic sequences of *DCL1* and *HYL1* with an ∼2-kb promoter region were cloned into the pENTR-D-TOPO vector (Invitrogen, Carlsbad, CA, USA). Next, site-directed mutagenesis was performed to introduce the *DCL1-13* point mutation and mutations in codons for S42 and S159 of HYL1. Finally, the LR reaction from the Gateway cloning system was used to subclone the *DCL1-13* and *HYL1* sequences into the pEarlyGate301 plasmid followed by *Agrobacterium* transformation (Earley et al. 2006). The pMIR157A:GUS, pMIR157C:GUS, pMIR161:GUS and pMIR163:GUS transgenic lines were obtained by floral dip transformation of WT plants (CLOUGH and BENT 1998). Regions ∼2 kb upstream from known TSS were cloned into the pENTR-D-TOPO vector (Invitrogen). Next, the Gateway cloning system was used to subclone promoter sequences into the pMDC163 plasmid followed by *Agrobacterium* transformation (Curtis and Grossniklaus 2003).

*Arabidopsis* plants were grown in soil (Jiffy-7 42 mm; Jiffy Products International AS, Stange, Norway) or on half-strength Murashige & Skoog (MS) medium with 0.8% agar square plates in growth chambers (Sanyo/Panasonic, no. MLR351, Japan) that had a day length of 16 h (150–200 µE/m^2^s), a constant temperature of 22°C and a humidity of 70%. Seeds were sterilized before sowing in 10% sodium hypochlorite in 70% EtOH solution.

### GUS staining for reporter line analysis

Fourteen-day-old *Arabidopsis* seedlings grown in half-strength MS medium were incubated in a staining solution containing 1 mM X-Gluc in 100 mM Na_3_PO_4_ (pH 7.2), 0.1% Triton X-100, 5 mM K_3_Fe(CN)_6_ and 5 mM K_4_Fe(CN)_6_ for 24 h at 37°C. Seedlings were then cleared in 70% ethanol for 2 d and mounted in 50% v/v glycerol before observation. After GUS staining, images were taken using a Leica M60 stereomicroscope.

### RNA isolation, cDNA synthesis and qPCR

Total RNA from 3-week-old or 14-day-old plants was isolated using TRIzol™ reagent (Invitrogen) and a Direct-zol RNA MiniPrep Kit (Zymo Research, Irvine, CA, USA). The RNA was then cleaned with Turbo™ DNase (Invitrogen) according to the provided protocol. Reverse transcription reactions were performed with SuperScript™ III Reverse Transcriptase (Invitrogen) and the oligo-dT primer. qPCR was performed with Power SYBR™ Green PCR Master Mix (Applied Biosystems) using a QuantStudio™ 7 Flex Real-Time PCR System (Applied Biosystems, Waltham, MA, USA). The expression levels were calculated using the 2^−ΔΔCt^ method. The Mann–Whitney *U* test was used for statistical analyses.

### Chromatin immunoprecipitation

ChIP was performed using nuclei isolated from crosslinked (1% formaldehyde) 21-day-old leaves, as described in Bowler et al. (2004), with minor modifications. Sonic and IP buffers were prepared as described in Kaufmann et al. (2010). Chromatin was sonicated at 4°C with a Diagenode Bioruptor Plus at high intensity for 30 min (30 s on/30 s off) to obtain a 200- to 300-bp DNA fragment size. Antibodies against total RNA Pol II (Abcam ab817) or HA antibodies were used with Dynabeads Protein G (Thermo Fisher Scientific, Waltham, MA, USA). Protein–DNA complexes were eluted from the beads as described in Rowley et al. (2013), and DNA was purified using a column-based method. DNA libraries were obtained using a MicroPlex Library Preparation Kit (Diagenode) and sequenced on a HiSeq HO 125 SE (Fasteris Plan-les-Ouates, Switzerland). qPCR was performed with Power SYBR™ Green PCR Master Mix (Applied Biosystems) using a QuantStudio™ 7 Flex Real-Time PCR System (Applied Biosystems).

### Analysis of ChIP-seq data

Raw reads were trimmed to 50 bp and aligned to the *Arabidopsis* genome (TAIR10) using Bowtie (with parameters −M1 −*n*2) (Langmead et al. 2009). Duplicate reads were removed by SAMtools (Li et al. 2009). The remaining sequences were extended to 200 bp according to the ChIP fragment length. Plots of RNA Pol II distribution were made using ngs.plot.r software (Shen et al. 2014). Genomic coordinates of *MIR* genes were taken from the mirEX 2.0 database (Zielezinski et al. 2015).

### PLA and *in situ* immunolocalization of HYL1 and RNA Pol II

The double immunodetection experiments were performed according to the protocol described in Bhat et al. (2020). For HYL1 localization, primary rabbit antibodies (Agrisera, Vannas, Sweden, no. AS06 136) diluted in 1:200 were used. After the double labeling assay, the slides were stained for DNA detection with Hoechst 33342 (Life Technolgies, Waltham, MA, USA) and mounted in ProLong Gold antifade reagent (Life Technologies).

### Coimmunoprecipitation assay followed by mass spectrometry

For each co-IP, nuclear extract was prepared as described earlier. Proteins were extracted from nuclear pellets by resuspending the pellets in nuclear lysis buffer [10% sucrose, 100 mM Tris-HCl (pH 7.5), 5 mM EDTA, 5 mM EGTA, 300 mM NaCl, 0.75% Triton X-100, 0.15% sodium dodecyl sulfate (SDS), 1 mM dithiothreitol (DTT) and 1× cOmplete™ EDTA-free protease inhibitor (Roche)] and sonicating for two cycles × 30 s on/30 s off using a Bioruptor® Plus (Diagenode, Seraing, Belgium). After removal of cell debris by centrifugation (5 min, 16,000×*g*, 4°C), the cleared supernatants were diluted one time using water containing 1× cOmplete™ EDTA-free protease inhibitor (Roche). Protein extract was incubated overnight with anti-HA antibodies (Roche). After overnight incubation, Dynabeads with protein G were added, and samples were incubated for 1 h. After incubation, beads were washed two times with low-salt buffer [20 mM Tris-HCl (pH 8), 2 mM EDTA, 150 mM NaCl, 1% Triton X-100 and 0.1% SDS], one time with high-salt buffer [20 mM Tris-HCl (pH 8), 2 mM EDTA, 500 mM NaCl, 1% Triton X-100 and 0.1% SDS], one time with LiCl buffer [10 mM Tris-HCl (pH 8), 1 mM EDTA, 250 mM LiCl, 1% NP40, 0.1% SDS and 1% sodium deoxycholate] and two times with TE [100 mM Tris-HCl (pH 8) and 1 mM EDTA]. Beads containing proteins were analyzed by mass spectrometry. Control IPs were performed in Col-0 using anti-HA antibodies.

Mass spectrometry analyses were performed by Institute of Biochemistry and Biophysics Polish Academy of Sciences, Warsaw, Poland. Magnetic beads containing proteins were suspended in 100 mM ammonium bicarbonate buffer, reduced using 100 mM DTT for 30 min at 57°C and alkylated in 50 mM iodoacetamide for 45 min at RT in the dark. In the next step, proteins were digested overnight using 100 ng/µl trypsin (Promega, Madison, WI, USA) at 37°C. Peptide mixtures were separated using a Nano-Ultra Performance liquid chromatograph coupled to an Orbitrap Velos mass spectrometer (Thermo Fisher Scientific). Peptides were identified with the Mascot algorithm (Matrix Science, London, UK) and searched against the TAIR10 database. The total number of MS/MS fragmentation spectra was used to quantify each protein from at least three independent biological replicates. Biological replicates consisted of plants of the same genotype grown on different dates and in different growth chambers. For the statistical analysis, we compared the data from three independent experiments for pHYL1:HYL1:HA. Statistical analysis was performed with the DESeq2 R package (Love et al. 2014).

### Coimmunoprecipitation assay followed by Western blot analysis

Crude protein extract was prepared from 0.5 g of ground tissue obtained from 14-day-old seedling. Proteins were extracted with lysis buffer [50 mM Tris-HCl (pH 8), 50 mM NaCl, 1% Triton X-100, 10 µM MG132 and 1× Protease Inhibitor Cocktail (Sigma P9599)]. After removal of cell debris by centrifugation (10 min, 16,000×*g*, 4°C), the cleared supernatant was used for IP using the µMACS HA Isolation Kit (Miltenyi Biotec, Bergisch Gladbach, Germany, no. 130-091-122) according to the manufacturer protocol. Anti-HA beads (150 µl) were used per IP. Proteins were detected by Western blot analyses with rabbit antibodies (Agrisera, AS06 136) diluted to 1:2,000 for HYL1, monoclonal mouse (Abcam, ab5408) diluted to 1:500 for RNA Pol II CTD repeat YSPTSPS (phospho S5) and monoclonal rat (Roche, no. 11867423001) diluted to 1:1,000 for HA epitope.

### Analysis of RNA-seq data

RNA-seq data from WT, *hyl1-2* and *se-3* were downloaded from a published dataset under the accession number ERP001616 (Manavella et al. 2012). RNA-seq data from *dcl1-9* and WT were downloaded from Wu et al. (2016). Raw reads were trimmed (first 20 nucleotides) with FASTX-Toolkit, adapters were removed using Trimmomatic and rRNA sequences were removed with Bowtie (Langmead et al. 2009, Bolger et al. 2014). Clean reads were aligned to the *Arabidopsis* TAIR10 reference genome using HISAT2 (Kim et al. 2015). The overall alignment rate was 98–99% for each sample. Next, the prepDE.py script was used to extract count information from the StringTie output, and the DESeq2 R package was used to find DEGs (Love et al. 2014). Gene ontology was performed using the clusterProfiler R package (Yu et al. 2012).

## Supplementary Material

pcad032_Supp

## Data Availability

The analyzed RNA-seq data were derived from sources in the European Nucleotide Archive repository under the accession number ERP001616 and in the National Center for Biotechnology Information (NCBI) Sequence Read Archive under the accession number SRP080774. The ChIP-seq data are deposited in the Gene Expression Omnibus under the accession number GSE222683. The generated mutants analyzed in this article will be shared on request to the corresponding author.
