# Supplementary material for: Hyponastic Leaves 1 Interacts with RNA Pol II to Ensure Proper Transcription of MicroRNA Genes
Source: Plant Cell Physiol. 2023 Apr 11;64(6):571–82. doi: 10.1093/pcp/pcad032 (PMC10269864; doi:10.1093/pcp/pcad032)
Supplement: pcad032_Supp [file pcad032_supp.zip › suppl_data/pcp-2022-e-00282-File006.pdf]

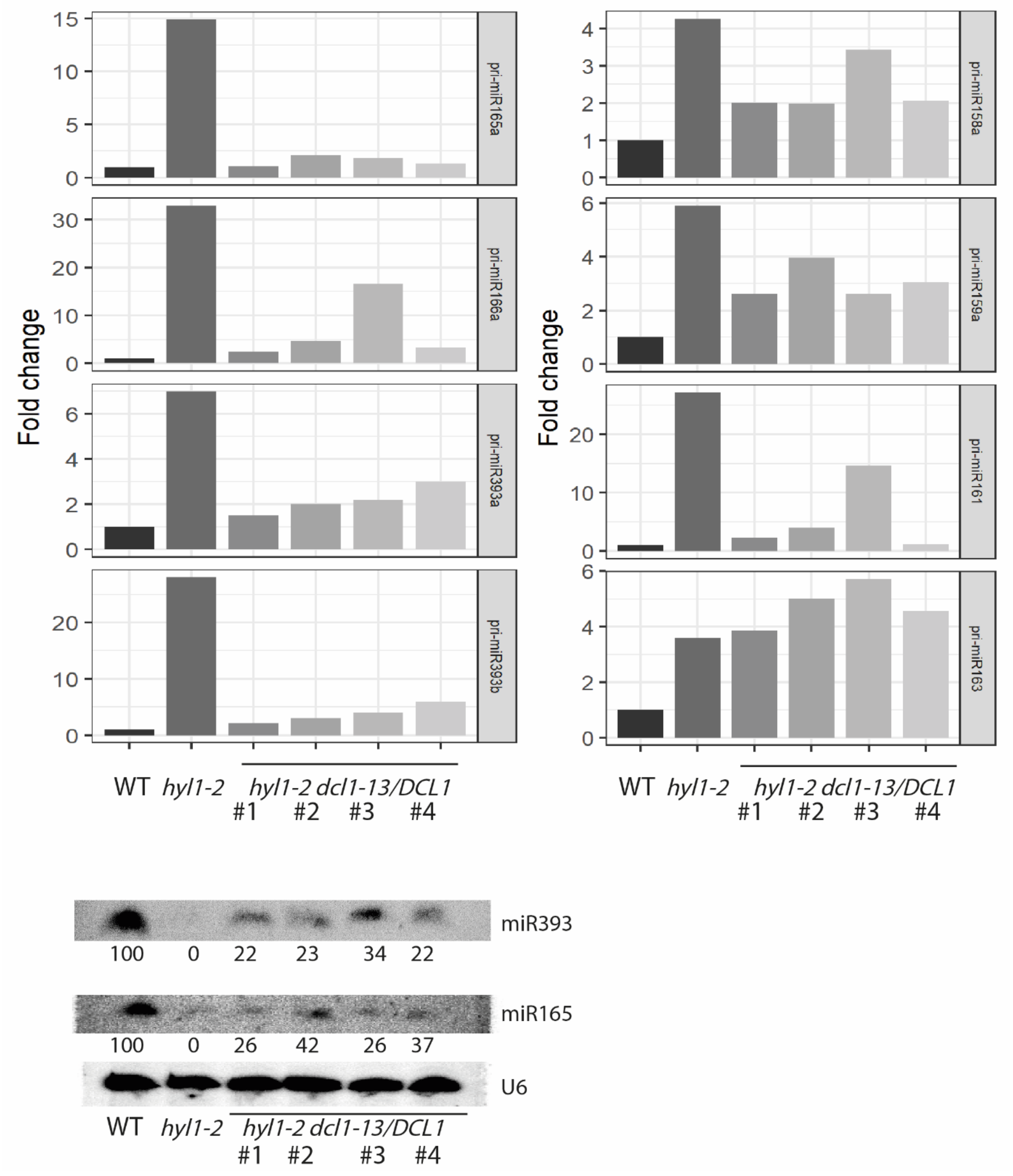

Figure S1. Characterization of *hyl1-2 dcl1-13/DCL1* plants. Upper panel: RT-qPCR analysis of 8 pri-miRNA levels in wild type, *hyl1-2* and plants from four independent *hyl1-2 dcl1-13/DCL1* transgenic lines (left). Bottom panel: Northern blot analysis of mature microRNA levels in wild type, *hyl1-2* and plants from four independent *hyl1-2 dcl1-13/DCL1* transgenic lines (right). U6 hybridization served as a positive control for northern blot hybridization

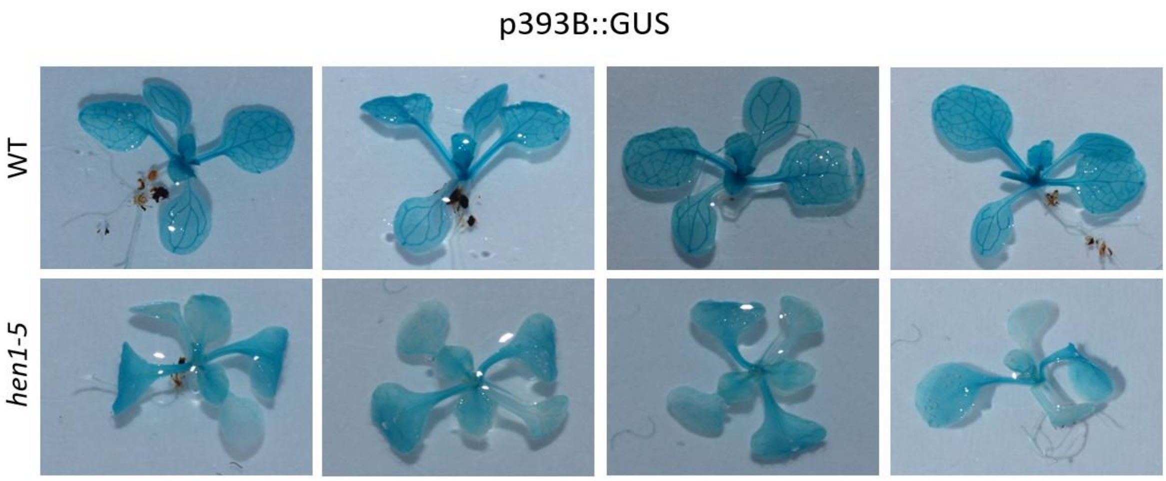

Figure S2. GUS staining of seedlings representing reporter p393b::GUS line in wild-type (top) and *hen1-5* (bottom).

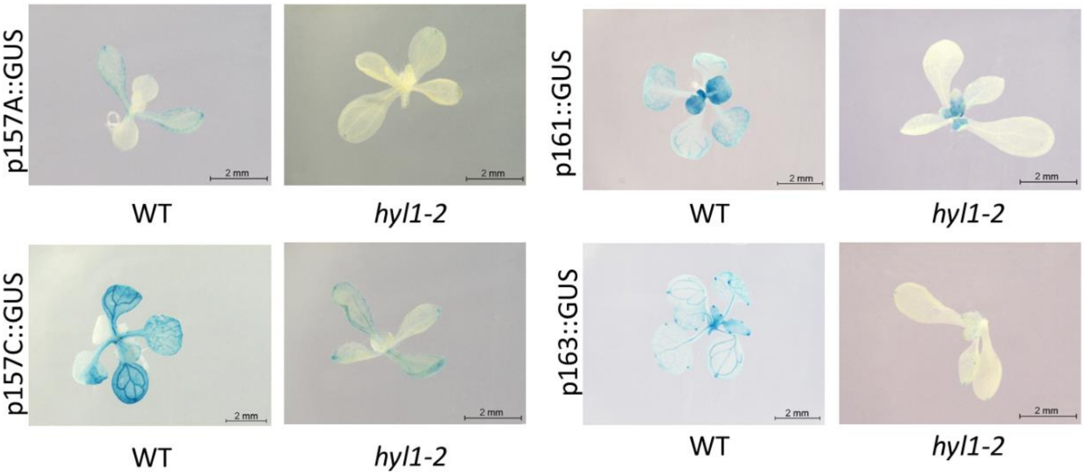

Figure S3. GUS staining of seedlings representing pMIR157A, pMIR157C, pMIR161 and pMIR163 reporter lines in wild-type and *hyl1-2*.

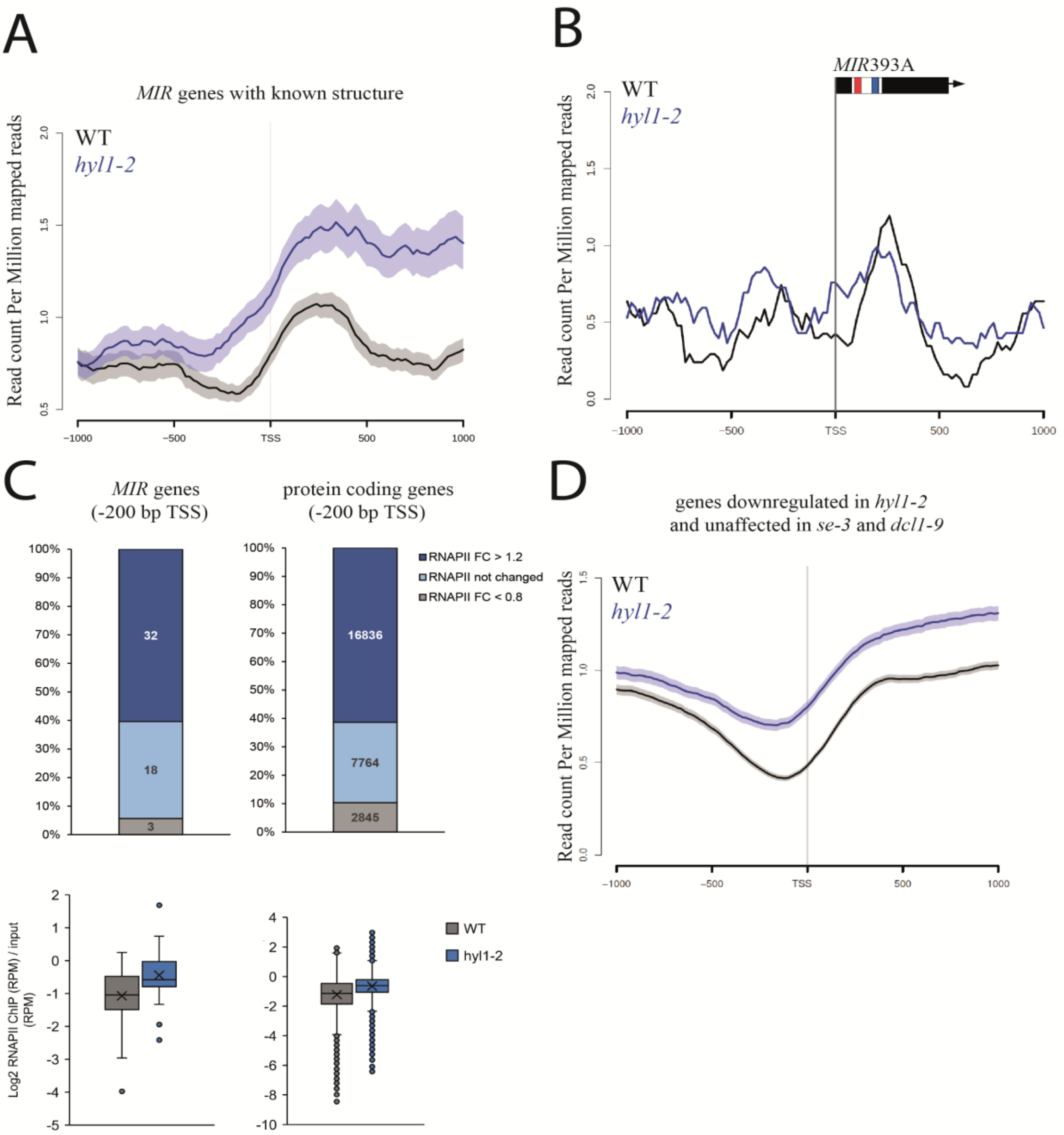

Figure S4. HYL1 is important for proper distribution of RNA Polymerase II along the *MIR393A* gene, and RNA Polymerase II distribution in *hyl1-2* is affected for *MIR* genes and protein coding genes. A) Accumulation of RNA Pol II in wild-type plants (black lines) and *hyl1-2* mutant plants (blue lines) for *MIR* genes with known structures **B)** Accumulation of RNA Pol II in wild-type plants (black lines) and *hyl1-2* mutant plants (blue lines) for the *MIR393A* gene, as determined by ChIP-seq. TSS, transcription start site. **C)** Comparison of RNA Polymerase II distribution at the region 200 bp upstream of transcription start site of *MIR* genes or protein coding genes**.** FC, fold change. RPM, reads per million. **D)** Accumulation of RNA Pol II in wild-type plants (black lines) and *hyl1-2* mutant plants (blue lines) for genes downregulated only in *hyl1-2* mutant plants (654 genes).

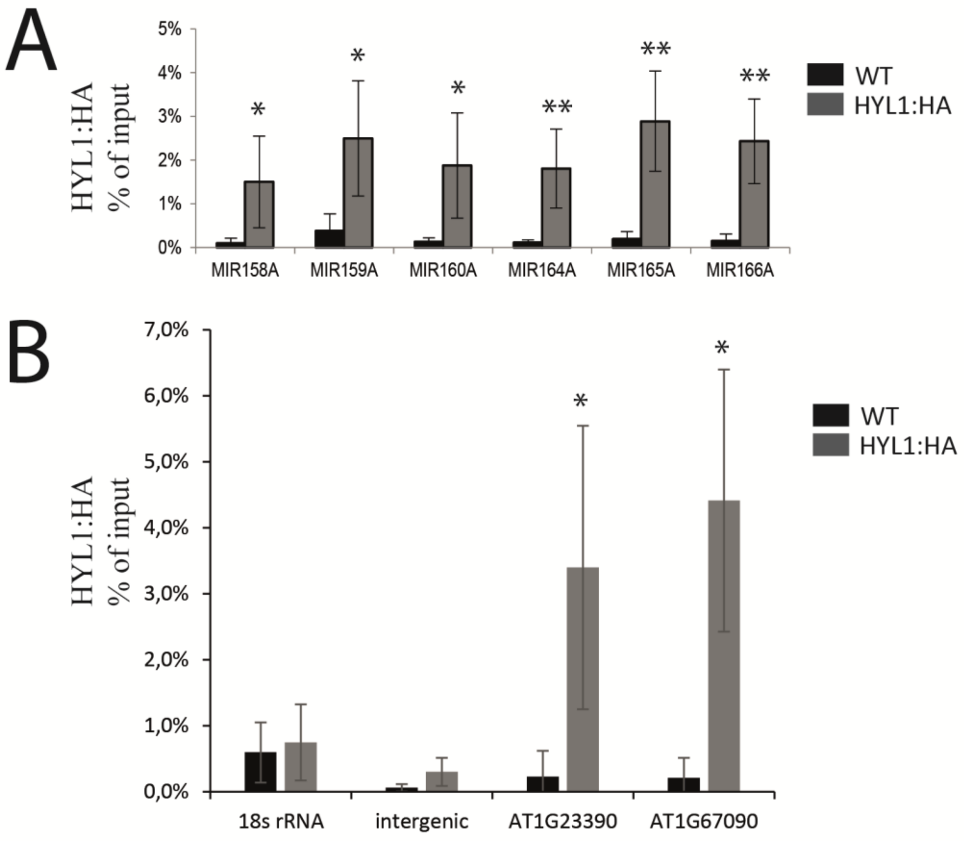

Figure S5. HYL1 is associated with chromatin. ChIP-qPCR analyses of HYL1 accumulation at the region 200 bp upstream of transcription start site of *MIR* genes (A) or protein coding genes (B). An asterisk represents the statistically significant enrichment of HYL1 in HYL1:HA compared to wild-type plants (WT).

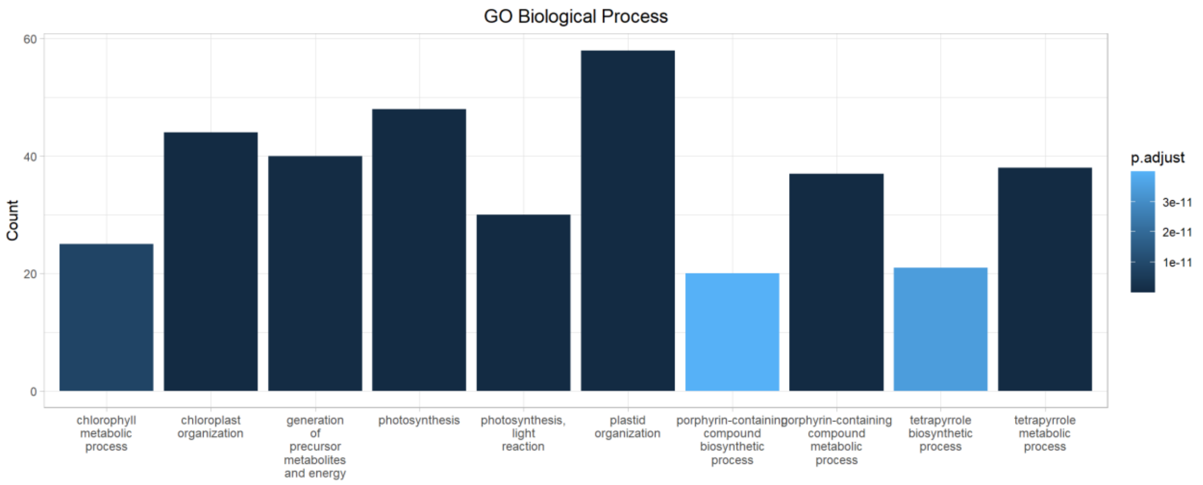

Figure S6. Genes showing downregulated expression in the *hyl1-2* mutant are involved in biological processes of 'plastid organization'. Gene ontology analysis of genes downregulated in the *hyl1-2* mutant in comparison to wild-type plants (group of 654 genes as in Figure 3C)**.**

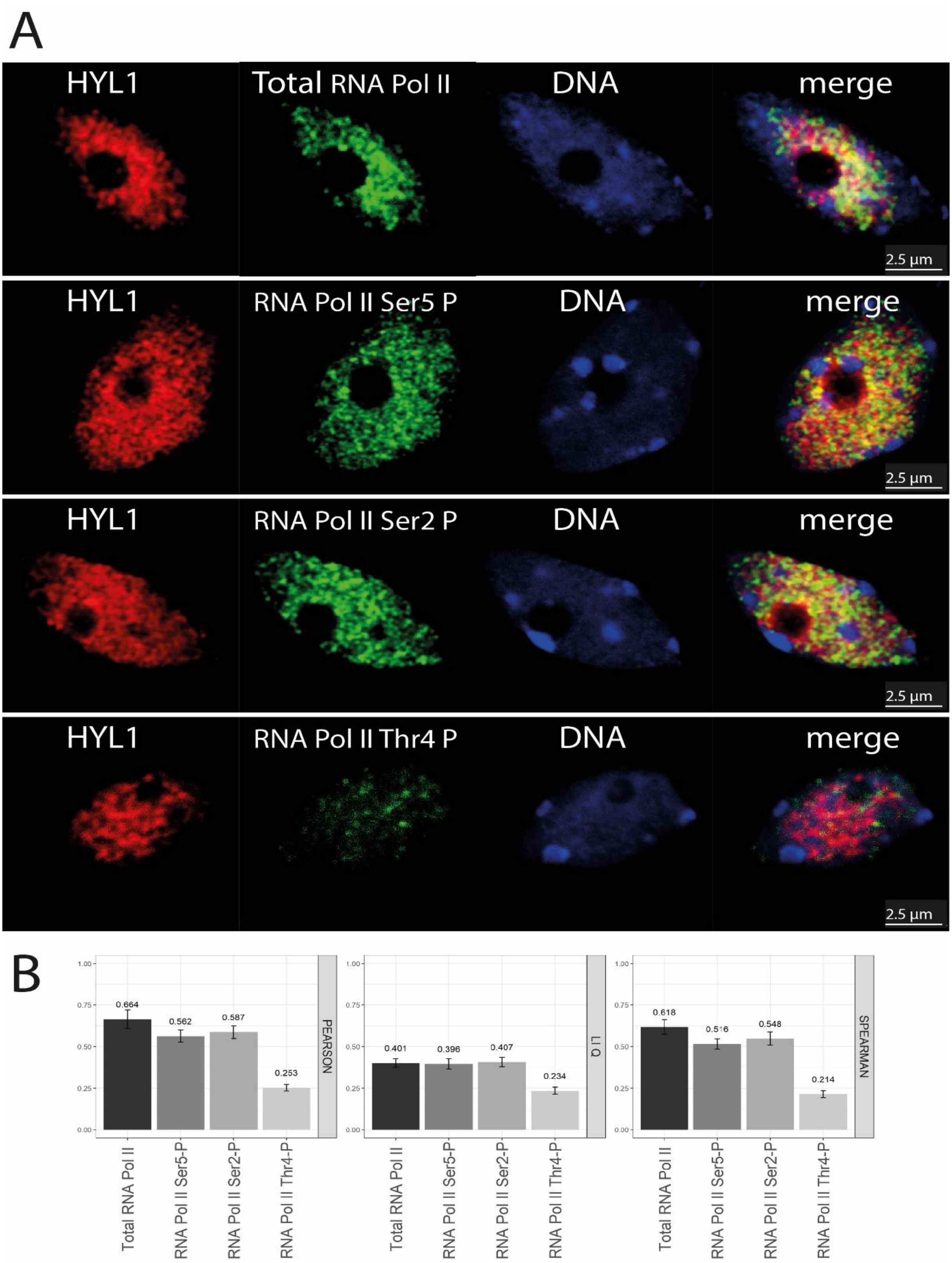

Figure S7. HYL1 colocalizes with RNA Polymerase II in wild-type plants. A) Nuclei from fixed cells, where HYL1 is shown in red, RNA Pol II in green and DNA in blue. The merged column shows all 3 channels. **B)** Colocalization scores of HYL1 and RNA Pol II calculated by three different approaches (Pearson, Spearman and LiQ).

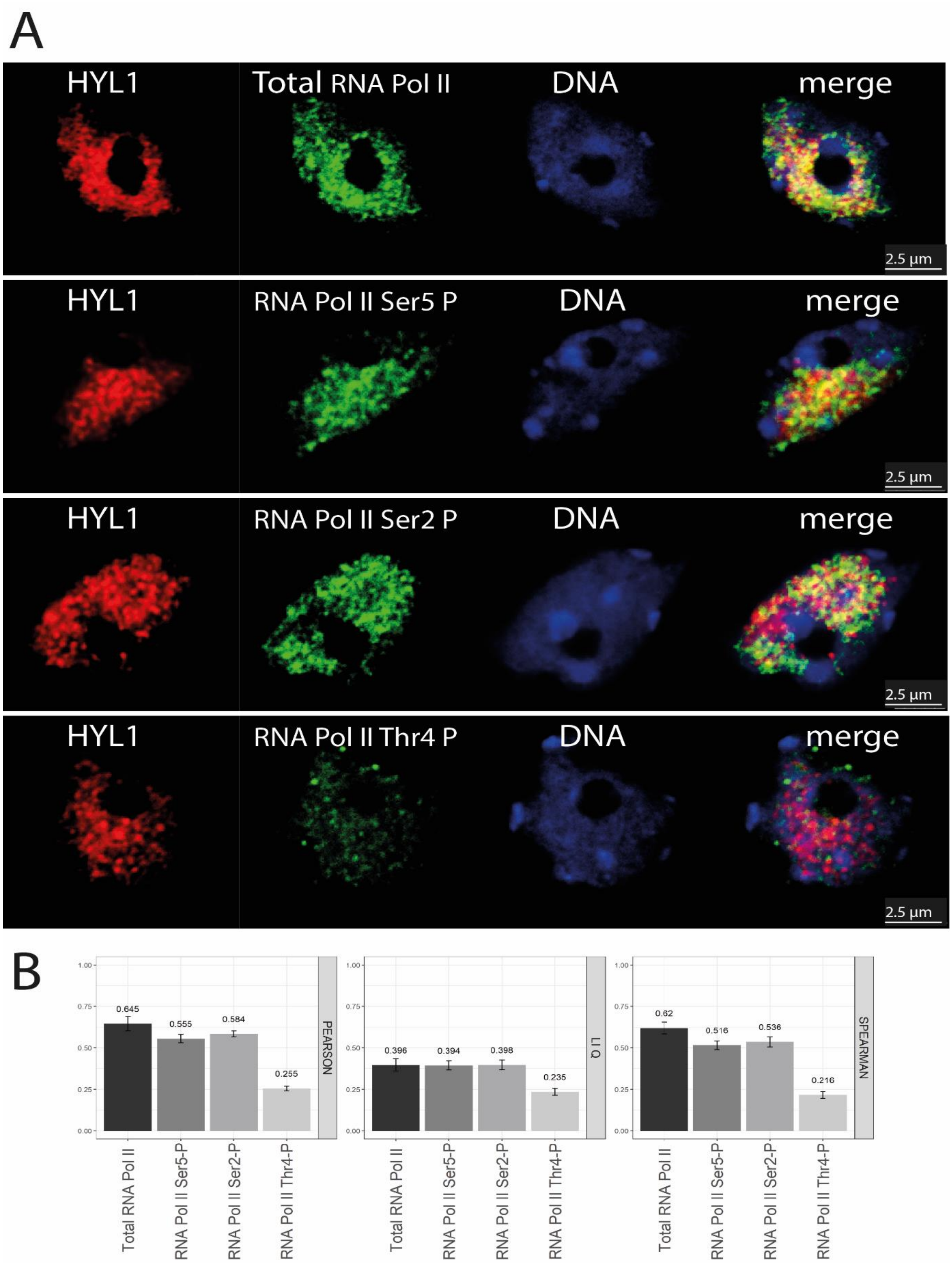

Figure S8. HYL1 colocalizes with RNA Polymerase II in *se-2* mutant plants. A) Nuclei from fixed cells, where HYL1 is shown in red, RNA Pol II in green and DNA in blue. The merged column shows all 3 channels. **A)** Colocalization scores of HYL1 and RNA Pol II calculated by three different approaches (Pearson, Spearman and LiQ).

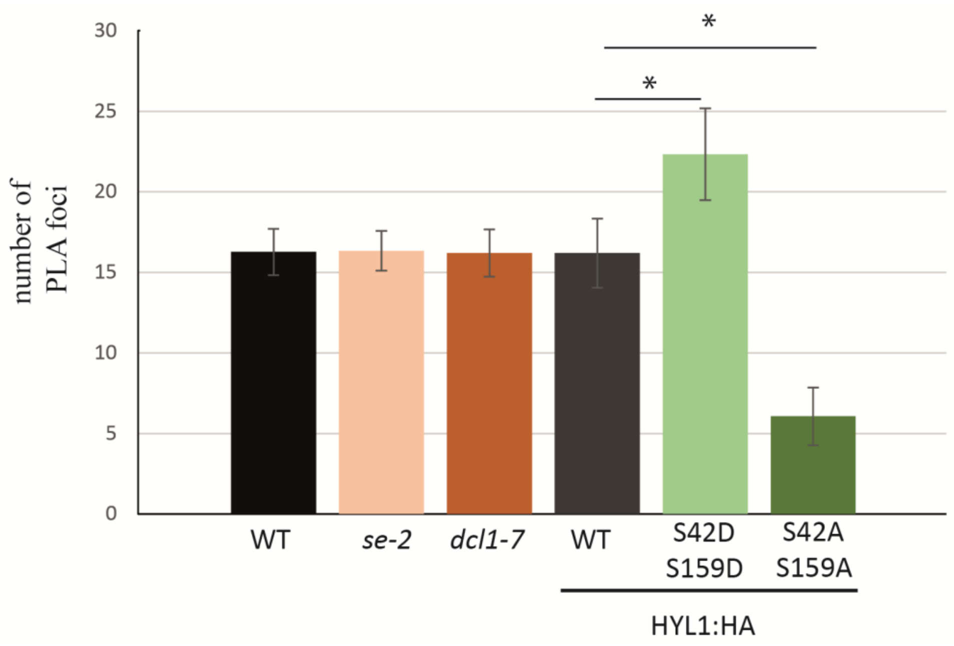

Figure S9. HYL1 interacts with RNA Pol II based on PLA assay. Quantification of the PLA foci presented in the Figure 4A and B. * pvalue <0.05, t-student test, at least 15 cells were analyzed for each examined genotype.

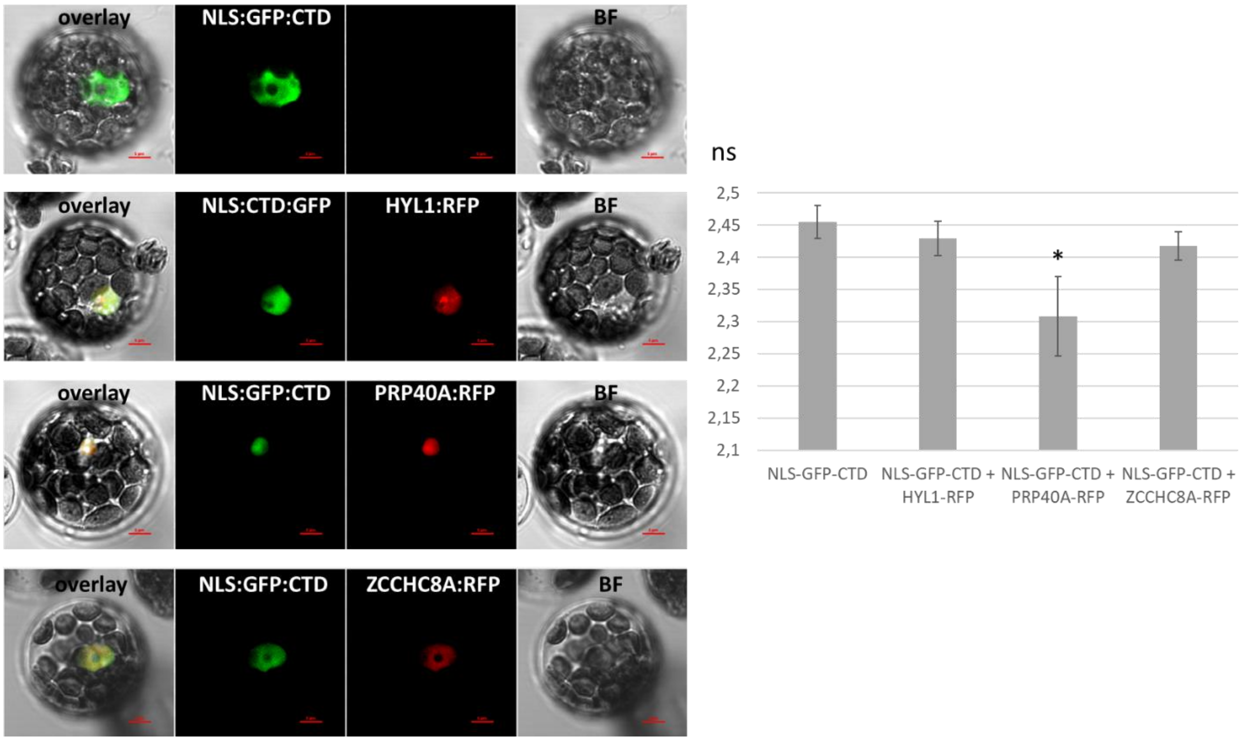

Figure S10. FRET-FLIM analysis of NLS-CTD with HYL1 in the nucleus. Transient co-expression of fluorescent proteins in Arabidopsis protoplasts. Scale bars are 5 µm, * pvalue <0.05, t-student test, BF - Bright Filed, ns - nanosecond.
